# A Human Conditionally Immortalized Proximal Tubule Epithelial Cell Line as a Novel Model for Studying Senescence and Response to Senolytics

**DOI:** 10.3389/fphar.2022.791612

**Published:** 2022-03-29

**Authors:** Yi Yang, Milos Mihajlovic, Floris Valentijn, Tri Q. Nguyen, Roel Goldschmeding, Rosalinde Masereeuw

**Affiliations:** ^1^ Utrecht Institute for Pharmaceutical Sciences, Division Pharmacology, Utrecht, Netherlands; ^2^ University Medical Center Utrecht, Department Pathology, Utrecht, Netherlands

**Keywords:** kidney fibrosis, conditionally immortalized proximal tubule epithelial cell, Bcl-2 family proteins, senescence-associated secretory phenotype (SASP), cell cycle arrest, apoptosis

## Abstract

Accumulating evidence suggests that senescence of kidney tubule epithelial cells leads to fibrosis. These cells secrete senescence-associated secretory phenotype (SASP) factors that are involved in diverse signaling pathways, influencing kidney fibrosis. Here, we investigated whether our previously established conditionally immortalized proximal tubule epithelial cell line overexpressing the organic anion transporter 1 (ciPTEC-OAT1) can be used as a valid *in vitro* model to study kidney senescence and senolytics response. CiPTEC-OAT1 proliferates rapidly at 33°C and exhibits a “senescence-like” arrest at 37°C, most likely due to suppression of SV40T expression and subsequent reactivation of the p53 and Rb pathways. To understand how permissive (33°C) and non-permissive (37°C) temperatures of the cell culture affect the senescence phenotype, we cultured ciPTEC-OAT1 for up to 12 days and evaluated the apoptosis and SASP markers. Day 0 in both groups is considered as the non-senescence group (control). Further, the potential of navitoclax, dasatinib, quercetin, and the combination of the latter two to clear senescent cells was evaluated. Maturation of ciPTEC-OAT1 at non-permissive temperature affected mRNA and protein levels of senescence markers. A remarkable upregulation in p21 gene expression was found in the non-permissive temperature group, whereas expression of Lamin B1 decreased significantly. SASP factors, including PAI-1A, IL-1β, CTGF, and IL-6 were upregulated, but no significant difference in Bcl-2 and Bcl-xl were found in the non-permissive temperature group. After culturing ciPTEC-OAT1 up to 12 days, cells in the non-permissive temperature group showed an upregulation in the apoptosis-associated proteins Bcl-2, BID, and Bax, and a downregulation in Mcl-1, Bad, Bak, and Bim at various time points. Further, Bcl-xl, Puma, Caspase 3, Caspase 7, and Caspase 9 showed initial upregulations followed by downregulations at later time points. The loss of Lamin B1, upregulation of SA-β-gal expression and increase in its activity, upregulation of p21 levels and downregulation of p53, along with the upregulation of SASP factors, confirmed that maturation at 37°C promotes senescence features. Finally, the senolytics response was evaluated by testing cell viability following exposure to senolytics, to which cells appeared dose-dependently sensitive. Navitoclax was most effective in eliminating senescent cells. In conclusion, culturing ciPTEC-OAT1 at 37°C induces a senescence phenotype characterized by increased expression of cell cycle arrest and anti-apoptosis markers, SASP factors, and responsiveness to senolytics treatment. Therefore, ciPTEC-OAT1 represents a valid model for studying kidney senescence by simply adjusting culture conditions.

## 1 Introduction

Renal fibrosis is the common end point for all progressive kidney diseases, which leads to kidney failure by an excessive accumulation of extracellular matrix (Boor et al., 2010). Accumulating evidence suggests that senescence of kidney tubule cells influences kidney fibrosis (Schafer et al., 2018). Senescence is a special form of permanent cell cycle arrest, which limits cellular proliferative life span. Some senescent cells can be cleared by immune cells, termed acute (short-term) senescent cells, while chronic (long-term) senescent cells keep accumulating and creating early senescence events, finally aggravating the pathology (Munoz-Espin and Serrano, 2014; Kobbe, 2019). Different hallmarks of senescence have been recognized which are involved in diverse signaling pathways, including apoptosis markers, senescence-associated secretory phenotype (SASP) factors, and cyclin-dependent kinase inhibitors (Hernandez-Segura et al., 2018).

Senescent cells show resistance to apoptosis (Childs et al., 2014) and accumulate dysfunctional mitochondria (Korolchuk et al., 2017). Mitochondrial outer membrane permeabilization (MOMP) is responsible for apoptosis in numerous cell death pathways (Chipuk et al., 2006). In the intrinsic apoptosis pathway, Bcl-2 and the caspase family proteins play important roles (Van Opdenbosch and Lamkanfi, 2019; Ngoi et al., 2020). The Bcl-2 family is divided into three main groups: anti-apoptotic (Bcl-2, Bcl-xl, and Mcl-1), pro-apoptotic (Bax and Bak), and pro-apoptotic BH3-only (Bim, Bid, Bad, and Puma) (Anantram and Degani, 2019). Senescent cells are known to be in a primed apoptotic state, triggered by the abnormal upregulation of anti-apoptotic and pro-apoptotic proteins (Fan et al., 2020). Caspase family proteins are downstream players of MOMP in the intrinsic apoptosis pathway (Shalini et al., 2015), and after the activation of the Bax-Bak-dependent MOMP, cytochrome C is released from the mitochondria stimulating caspase-9 activation and its downstream executioners, caspases-3 and -7, to initiate apoptosis (Van Opdenbosch and Lamkanfi, 2019).

Cell cycle arrest is another typical characteristic of senescent cells, which is largely mediated through activation of either one or both p53/p21^CIP1/WAF1^ (p21) and p16^Ink4a^ (p16)/pRb pathways (Kumari and Jat, 2021). p53/p21 is activated during DNA damage response, resulting in a p21-dependent G0/G1 cell-cycle arrest (Ceccaldi et al., 2012; Ou and Schumacher, 2018). On the other hand, p16 inactivates Retinoblastoma 1 (pRb) thereby inhibiting the action of the cyclin dependent kinases, leading to G1 cell cycle arrest (Rayess et al., 2012). Both p53/p21 and p16/pRb pathways are independent in senescence induction. Acute DNA damage causes a cell cycle arrest via the p53/p21 pathway, while chronic DNA damage followed by the induction of the p16/pRB pathway maintains cell cycle arrest and senescence (Sperka et al., 2012). As a key mediator of cell-cycle arrest, p21 also shows a p53-independent upregulation according to some research (Zhang et al., 2011; Ruan et al., 2020). Furthermore, p53 is also involved in the apoptosis process, as described previously (Rufini et al., 2013; Ou and Schumacher, 2018).

SASP factors are related to a DNA damage response and are generally proinflammatory and/or profibrotic compounds including numerous cytokines (e.g., IL-6 and IL-8), growth factors (e.g., TGF-β and CTGF), chemokines (e.g., CCL2), and matrix-metalloproteinases (e.g., MMP-1 and MMP-3) (Hernandez-Segura et al., 2018; Birch and Gil, 2020). Those proteins induce or maintain senescence through different pathways, contributing to kidney fibrosis (Docherty et al., 2019). Current efforts are focused on clearing senescent cells as a treatment option for prevention of kidney fibrosis development and progression. Senolytics represent a good option as they can selectively eliminate senescent cells participating in senescence associated pathways by interfering with anti- and pro-survival signaling (Zhu et al., 2015). However, suitable cell models are required to evaluate senescence development in kidney tubule epithelial cells and their response to senolytics.

We previously developed a conditionally immortalized proximal tubule epithelial cell line overexpressing the organic anion transporter 1 (ciPTEC-OAT1) and applied it successfully for pharmacological and toxicological investigations, including drug disposition and interaction studies (Wilmer et al., 2010; Nieskens et al., 2016). OAT1 is a first step in the elimination of organic anions in humans and is responsible for the uptake of many anionic (waste) products in kidney proximal tubules (Pou Casellas et al., 2021). Since the expression of OAT1 is rapidly lost when culturing (primary) PTEC *in vitro*, OAT1 was stably expressed in ciPTEC by lentiviral transduction. This cell line now allows prediction of drug-induced nephrotoxicity and drug-drug interactions of organic anions *in vitro* (Nieskens et al., 2016). OAT1 is also involved in the uptake of uremic toxins, known to participate in the uremic syndrome typical of chronic kidney disease (Nigam and Bush, 2019). Since senescence is a key factor contributing to chronic kidney disease, uremic toxins might play a role in this process as well.

CiPTEC was created by means of a temperature sensitive mutant U19tsA58 of SV40 large T antigen (SV40T) and the essential catalytic subunit of human telomerase (hTERT), to keep the characteristics of primary cells (Wilmer et al., 2010). Temperature-sensitive SV40T allows cells to proliferate at the permissive low temperature of 33°C but induces a proliferation block that resembles senescence at a non-permissive temperature of 37°C (Larsson et al., 2004; Wilmer et al., 2010). The hTERT maintains telomere length, preventing replicative senescence induced by telomere shortening (Bodnar et al., 1998). Some studies already showed a relation between senescence and SV40T conditional models, because both pRb and p53 are activated by SV40T at the non-permissive temperature leading to a senescence-like arrest in the cells (Larsson et al., 2004; Brookes et al., 2015). Therefore, we hypothesized that ciPTEC-OAT1 exhibits a senescence phenotype when cultured at non-permissive temperatures that can be used to study senescence development in kidney tubule epithelial cells and their response to senolytics. In the present study, we evaluated apoptosis markers and other common senescence markers in ciPTEC-OAT1 cultured at permissive and non-permissive temperatures at different time points, to investigate whether these cells can be implemented as a valid *in vitro* model to study kidney senescence. Day 0 in both groups is considered as the non-senescence group (control). Finally, the senolytics response was detected by means of cell viability assessment and senescence-associated β-galactosidase (SA-β-gal) activity.

## 2 Materials and Methods

### 2.1 Quantitative Real-Time PCR

CiPTEC-OAT1 cells were seeded into 6-well format plates and grown at 33°C; then half of the plates were transferred to 37°C and cultured for up to 7 d. Afterward, cells were lysed in Trizol (Thermo-Fisher, Massachusetts, United States) followed by 5 min centrifugation at 4°C. After RNA isolation, RNA quantity was determined using Nanodrop 2000 (Thermo-Fisher, Massachusetts, United States). For RNA analysis, a cDNA library was synthesized using 3 μg RNA per sample with SuperScript III reverse transcriptase (Thermo-Fisher, Massachusetts, United States). Samples were mixed with TaqMan Gene Expression Assays (Table 1) and run on a ViiA 7 real-time PCR system (Applied Biosystems, California, United States). TATA-box binding protein (TBP) was used as an internal reference gene. Samples were run in duplicate and H_2_O samples were used to control for potential contamination of reaction. The ΔΔCT method was used to calculate relative expression levels.

**TABLE 1:** Primers used for real-time polymerase chain reaction.

Gene	Taqman gene expression assay
TBP	Hs00427620_m1
Lamin B1	Hs01059210_m1
p21 (CDKN1A)	Hs00355782_m1
CTGF	Hs00170014_m1
PAI-1(SERPINE1)	Hs00167155_m1
IL-1β	Hs01555410_m1
IL-6	Hs00174131_m1
BCL-2	Hs00608023_m1
Bcl-xl (BCL2L1)	Hs00236329_m1

### 2.2 ciPTEC-OAT1 Maturation Process

CiPTEC-OAT1 were grown and expanded at 33°C. Following the seeding, cells were either kept at a permissive temperature of 33°C or incubated for a desired time up to 12 d at a non-permissive temperature of 37°C. The culture medium and cell lysate were collected on Day 0, 3, 6, 9, and 12, and used for the assessment of senescence markers and phenotype.

The ciPTEC-OAT1 cell line was cultured as reported previously (Mihajlovic et al., 2017). Briefly, cells were cultured in phenol-red free DMEM-HAM’s F12 medium (Gibco, Life Technologies, Paisley, United Kingdom) supplemented with 10% (v/v) fetal calf serum (FCS) (Greiner Bio-One, Alphenaan den Rijn, the Netherlands), 5 μg/ml insulin, 5 μg/ml transferrin, 5 μg/ml selenium, 35 ng/ml hydrocortisone, 10 ng/ml epidermal growth factor (EGF), and 40 pg/ml tri-iodothyronine to form complete culture medium, up to a maximum of 60 passages. Cells were cultured at 33°C and 5% (v/v) CO_2_ to allow proliferation. Cells were grown up to 90% confluence at 33°C, then transferred for 7 d or 9 d at 37°C, 5% (v/v) CO_2_ for maturation, refreshing the medium every other day.

### 2.3 Senescence and Senolytics Response

#### 2.3.1 Cell viability Assay

Cell viability was measured using PrestoBlue® cell viability reagent (Thermo Scientific, Vienna, Austria). CiPTEC-OAT1 cells were seeded into 96-well format plates at a density of 63,000 per well, cultured for 24 h at 33°C and matured for 9 d at 37°C. Matured cells (37°C) were exposed to 100-μl medium with different concentrations of navitoclax, dasatinib, quercetin, or dasatnib and quercetin combinations. Senolytics were obtained from MedchemExpress, the Netherlands. All experiments were performed in a 96-well plate setup in triplicate with a minimum of three independent experiments.

#### 2.3.2 SA-β-Gal Staining Assay

CiPTEC-OAT1 cells were seeded into 12-well format plates, grown at 33°C, then transferred to 37°C for maturation and culturing for 9 d. The cells matured at 37°C for 0 d and 9 d were exposed to 1-ml medium with different concentrations of navitoclax, dasatinib, or dasatnib and quercetin combinations. The SA-β-gal-positive cells were detected using Senescence Detection Kit (ab65351, Abcam, United Kingdom), and evaluated for blue colorization using an optical microscope (200x magnification).

### 2.4 Western Blot

CiPTEC-OAT1 cells were lysed in ice-cold RIPA Lysis Buffer (Thermo Scientific, Vienna, Austria) for 30 min followed by 20 min centrifugation at 4°C and obtained protein samples were quantified by BCA Protein Assay Kit (Thermo Scientific, Vantaa, Finland). Proteins were loaded and separated on 14–20% acrylamide gradient SDS gels (Bio-Rad Laboratories, Hercules, CA), transferred to PVDF membranes (Bio-Rad Laboratories, Hercules, CA) in appropriate transferring conditions (25V, 7 min). The membranes were blocked in 5% skim milk-TBST for 2 h and incubated with the primary antibody overnight at 4°C and anti-rabbit (1:3000, Dako, P0448, United States) or anti-mouse (1:3,000, Dako, P0260, United States) secondary antibodies for 1 h at room temperature. The membrane was exposed to Clarity Western ECL Blotting Substrate following manufacturer’s instructions (Bio-Rad Laboratories, Hercules, CA) then imaged using the ChemiDoc™ MP Imaging System (Bio-Rad Laboratories, Hercules, CA) to detect the protein bands, which were quantified using ImageJ software (version 1.53c, National Institutes of Health, United States).

The following proteins were detected by Western blotting: Bcl-2, Bcl-xl, Mcl-1, Bad, Bak, Bim, BID, Bax, Puma, Caspase-3, Caspase-7, Caspase-9, p53, p21, and β-gal, for which primary antibodies were purchased from Cell Signaling Technology (United Kingdom). LaminB1 primary antibody was purchased from Abcam (United Kingdom). The dilution of all primary antibodies was 1:1000.

### 2.5 ELISA

Cell culture supernatants were centrifuged for 10 min, 240 x g, 4°C, and stored at −20°C. To determine the concentration of SASP factors in the culture supernatants, the ELISA Kits of IL-6 (88-7066-88, Invitrogen, Carlsbad, CA), IL-8 (88-8086-88, Invitrogen, Carlsbad, CA), CTGF (DY9190-05, R&D System, United Kingdom), TNF-α (88-7346-88, Invitrogen, Carlsbad, CA), and TGF-β1(88-8350-88, Invitrogen, Carlsbad, CA) were used according to the manufacturer’s instructions.

### 2.6 Statistics

All data analysis and statistics were performed using the GraphPad Prism (version 8.3.0; GraphPad software, La Jolla, CA), and expressed as mean ± SEM. For comparison of two groups at different temperature and different time points, two-way ANOVA was used followed by Sidak’s multiple comparison test. To compare multiple groups in the same condition, one-way ANOVA was used followed by Dunnett’s multiple comparison test. *p* < 0.05 was considered significant. Cell viability was expressed as inhibitory constants at 50% of control viability levels (IC_50_ values), which were calculated by plotting log senolytics concentration vs. viability following background subtraction. Nonlinear regression with a variable slope constraining the bottom to 0 was used to fit the normalized data. For cell viability at Day 9 compared to Day 0 evaluations at different concentrations, two-way ANOVA was used followed by Sidak’s multiple comparison test. For cell viability of increasing concentrations of dasatinib combined with quercetin compared to dasatinib alone, a two-way ANOVA was used followed by Dunnett’s multiple comparisons test.

## 3 Results

### 3.1 Growing ciPTEC-OAT1 at Non-Permissive Temperature (37°C) Induces Expression of Senescence-Associated Genes

**FIGURE 1:**
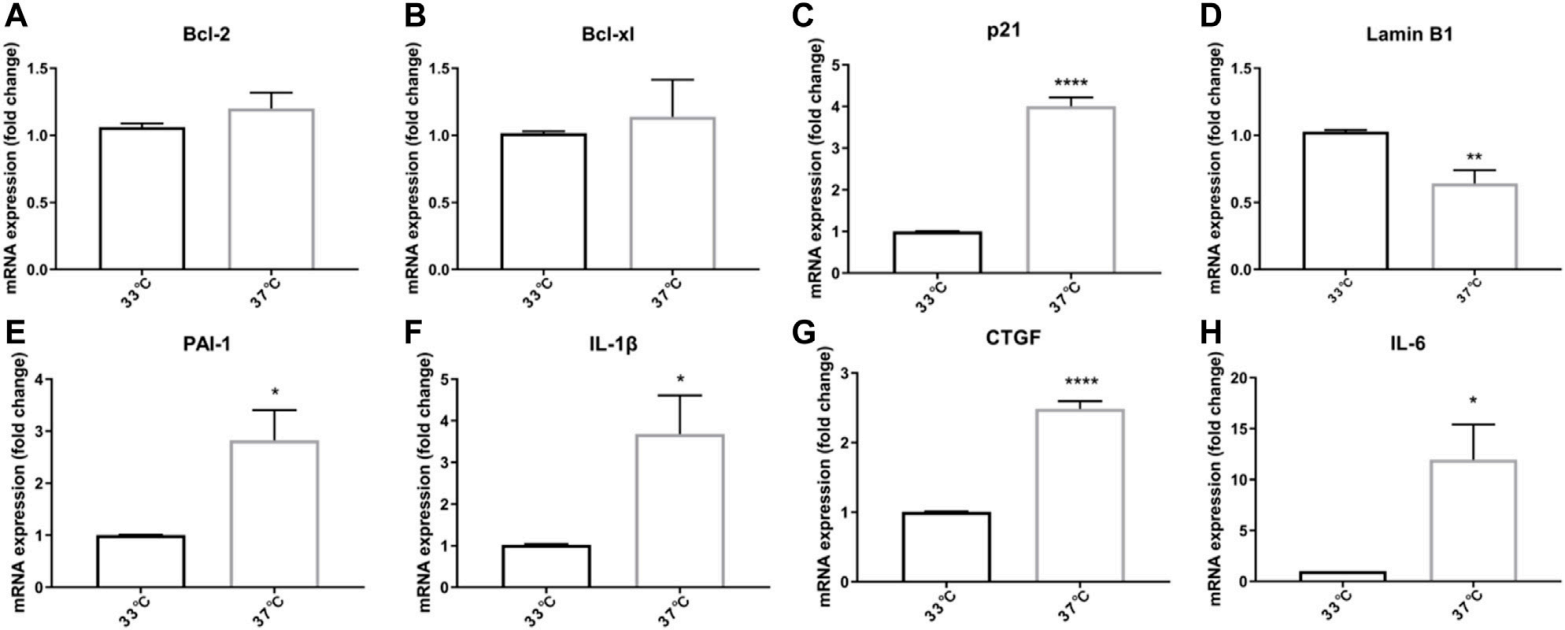
Growing ciPTEC at a non-permissive temperature (37°C) induces expression of senescence-associated genes. **(A)** Bcl-2. **(B)** Bcl-xl. **(C)** p21. **(D)** LaminB1. **(E)** PAI-1. **(F)** IL-1β. **(G)** CTGF. **(H)** IL-6. Four independent experiments in triplicate were performed. ^*^ *p* < 0.05, ^**^ *p* < 0.01, and ^****^ *p* < 0.0001 (unpaired *t* test). For clarity, only one-sided error bars are shown.

To understand if non-permissive temperature leads to cellular senescence, we compared expression of several senescence markers on mRNA level after culturing ciPTEC-OAT1 for 7 d at permissive (33°C) and non-permissive temperature (37°C). Compared to the permissive temperature group, no significant difference in the mRNA levels of Bcl-2 and Bcl-xl was observed (Figures 1A,B). On the other hand, a significant upregulation of p21 mRNA levels was found in the non-permissive temperature group compared to the permissive temperature group (*p* < 0.0001; Figure 1C). Furthermore, Lamin B1 mRNA levels decreased when cells were cultured at non-permissive temperature (*p* < 0.01; Figure 1D). SASP factors including PAI-1, IL-1β, CTGF, and IL-6 were all upregulated in the non-permissive temperature group (*p* < 0.05, *p* < 0.05, *p* < 0.001, and *p* < 0.05, respectively; Figure 1E-H).

### 3.2 Maturation at Non-Permissive Temperature of 37°C Affects Protein Levels of Apoptosis-Associated Markers in ciPTEC-OAT1

**FIGURE 2:**
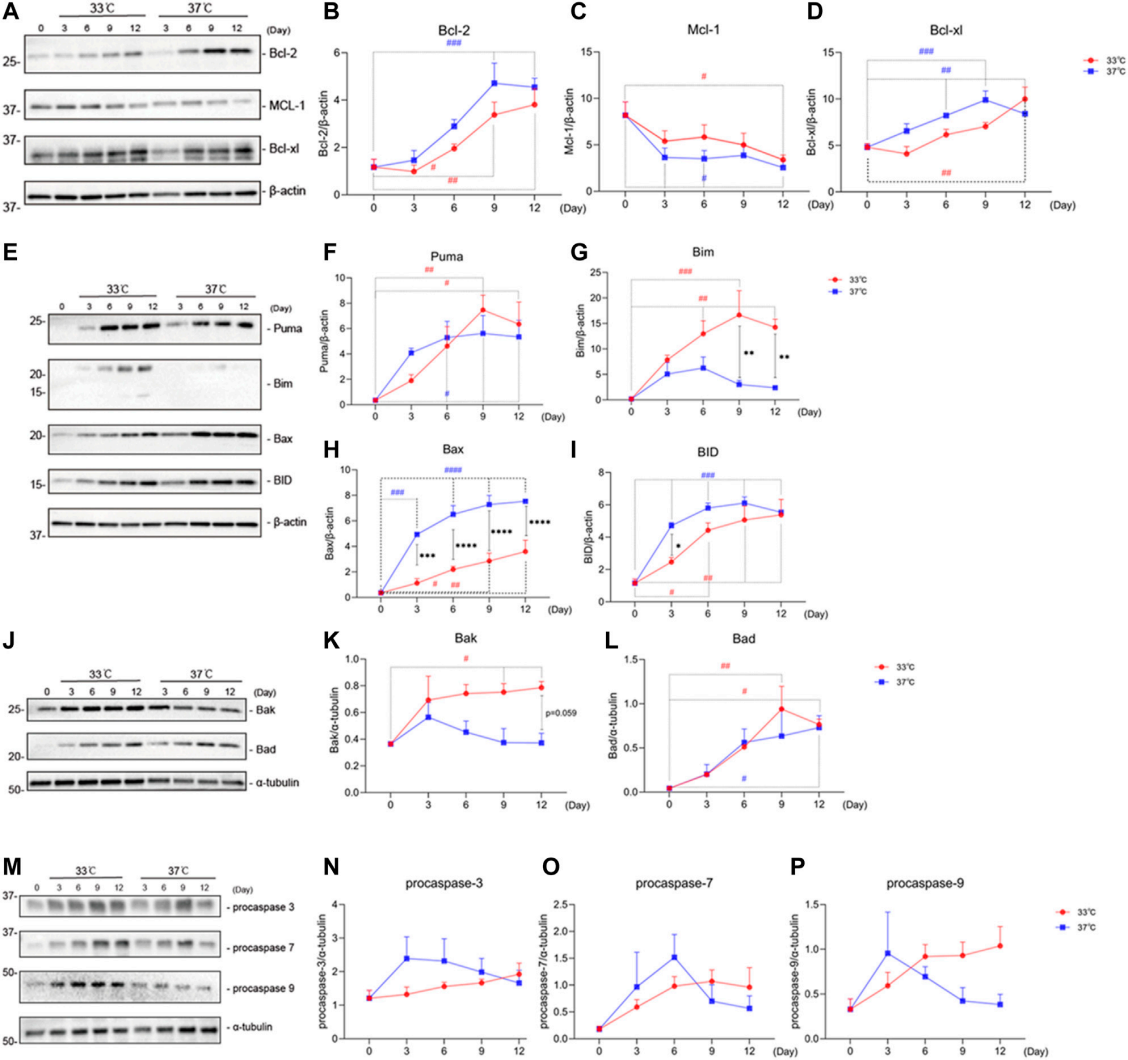
Maturation at a non-permissive temperature of 37°C affects protein levels of apoptosis-associated markers in ciPTEC-OAT1. **(A)** Representative Western blots showing expression of anti-apoptotic proteins Bcl-2, Bcl-xl, and Mcl-1 belonging to the Bcl-2 family. **(B),(C),** and **(D)** Relative expression of Bcl-2, Bcl-xl, and Mcl-1 over time (Day 0 to Day 12) at both permissive and non-permissive temperatures of 33°C and 37°C, respectively. **(E)** and **(J)** Representative Western blots of the pro-apoptotic proteins’ expression (Puma, Bim, Bax, and BID shown in **(E)**, Bak and Bad shown in **(J)** of Bcl-2 families. **(F)–(L)** Relative expression levels of Puma, Bim, Bax, BID, Bak, and Bad over time at 33°C and 37°C. **(M)** Representative Western blots showing expression of procaspase-3, procaspase-7, and procaspase-9. **(N),(O),** and **(P)** Relative expression of procaspase-3, procaspase-7, and procaspase-9 at different time points, at 33°C and 37°C. Protein expression levels were normalized against α-tubulin or β-actin and expressed as mean ± SEM. Three independent experiments in triplicate were performed. *p < 0.05, ^**^ *p* < 0.01, ^***^ *p* < 0.001, ^****^ *p* < 0.0001 (expression levels at 37°C compared to 33°C; two-way ANOVA, Sidak’s multiple comparison test). ^#^ *p* < 0.05, ^##^ *p* < 0.01, ^###^ *p* < 0.001, ^####^ *p* < 0.0001 (expression levels at Day 3, 6, 9, or 12 compared to Day 0, at 33°C or 37°C; one-way ANOVA, Dunnett’s multiple comparison test). Day 0 in both groups is considered as the non-senescence group (control). For clarity, only one-sided error bars are shown.

Senescent cells are characterized by an anti-apoptotic profile (Munoz-Espin and Serrano, 2014). To test whether maturation at the non-permissive temperature of 37°C would affect protein levels of apoptosis-associated markers in ciPTEC-OAT1, both anti- (Figures 2A–D) and pro-apoptotic (Figures 2E–P) protein markers were evaluated.

Representative Western blots and quantitative relative expression data indicate that there is an increased expression of Bcl-2 (Figure 2B) over time regardless of the temperature. However, there is a trend of different expression levels between the two temperature conditions at each day. The protein levels of Mcl-1 on the other hand (Figure 2C) decreased with time at all conditions tested, compared to Day 0, the non-senescence group. Bcl-xl (Figure 2D) levels showed an upregulation at both 33 and 37°C at almost all time points.

With respect to the pro-apoptotic proteins, we observed that all Bcl-2 family members tested, including Puma (Figure 2F), Bax (Figure 2H), BID (Figure 2I), and Bad (Figure 2L) showed an increase at both permissive (33°C) and non-permissive (37°C) groups at different time points. Significant differences in expression levels between 37 and 33°C groups were observed for Bax on Day 3 through Day 12, as well as for Bad on Day 3. Unlike other pro-apoptotic proteins among the Bcl-2 family members, Bim (Figure 2G) showed an upregulation at the permissive temperature at various time points, while an initial upregulation on Day 3 through Day 6 followed by a downregulation on Day 9 and Day 12 at the non-permissive temperature. In addition, the protein levels of Bim at 37°C were lower than at 33°C on Day 9 and Day 12. On the other hand, Bak (Figure 2K) showed an increased expression at 33°C on Day 3 through Day 12, while a decrease in protein levels was observed at 37°C starting from Day 3.

For the pro-apoptotic caspases, procaspase-3 (Figure 2N), procaspase-7 (Figure 2O), and procaspase-9 (Figure 2P), we found a non-significant trend for overtime upregulation at a permissive temperature, while at a non-permissive temperature, after an initial upregulation, there was a non-significant trend of downregulation at later time points, starting at Day 3 for procaspases-3 and -9, and at Day 6 for procaspase-7.

### 3.3 Maturation of ciPTEC-OAT1 at Non-Permissive Temperature Affects Expression Levels of Common Senescence Makers

**FIGURE 3:**
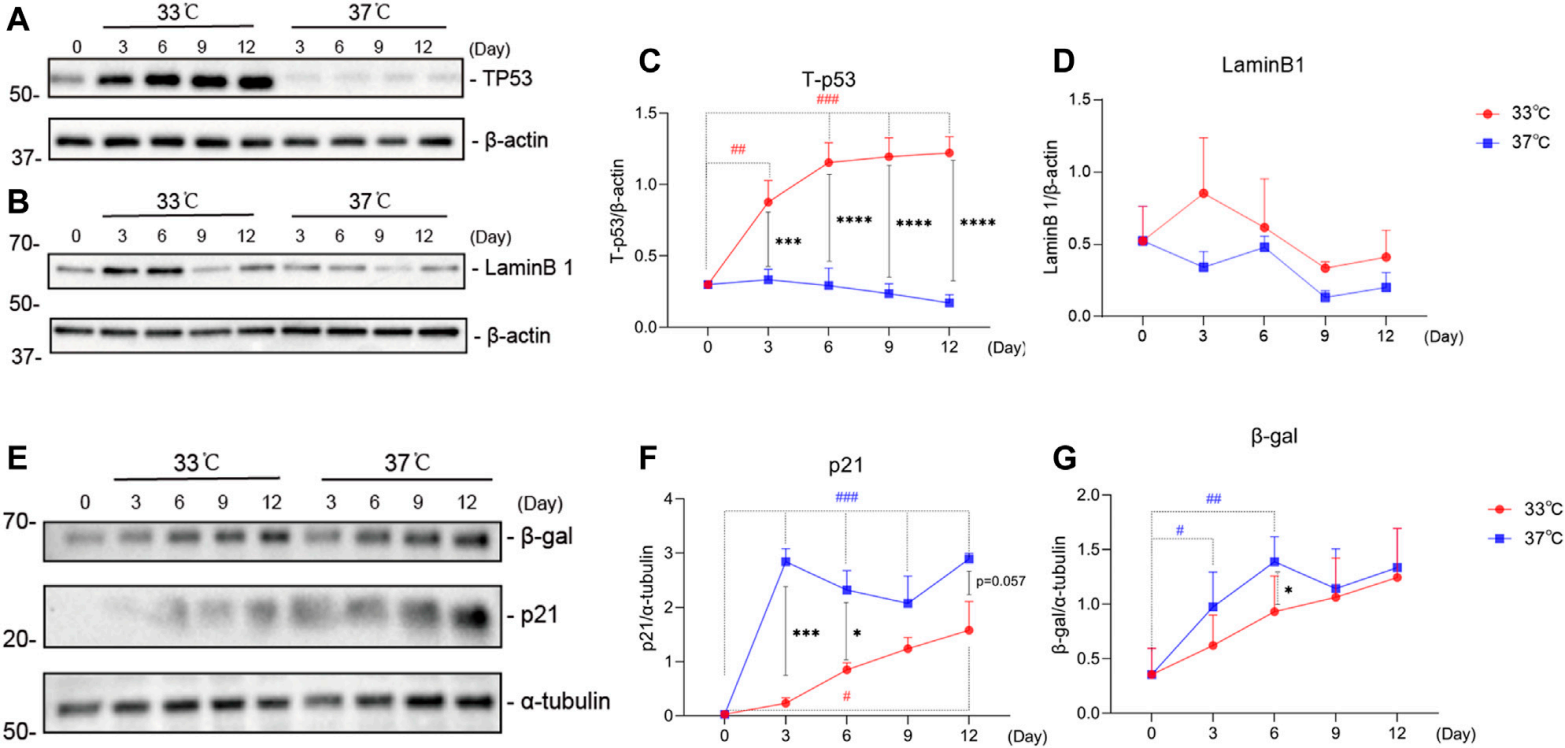
Maturation at a non-permissive temperature of 37°C affects common senescence markers expression in ciPTEC-OAT1. Representative Western blots showing expression of **(A)** total-p53 (Tp53), **(B)** LaminB1, **(E)** p21 and β-gal. **(C),** **(D),** **(F),**and **(G)** Relative expression levels of **(C)** total-p53, **(D)** LaminB1, **(F)** p21, and **(G)** β-gal over time (Day 0 to Day 12), at both permissive (33°C) and non-permissive temperatures (37°C). Protein expression levels were normalized against α-tubulin or β-actin and expressed as mean ± SEM. Three independent experiments in triplicate were performed. *p < 0.05, **p < 0.01, ***p < 0.001, ****p < 0.0001 (expression levels at 37°C compared to 33°C; two-way ANOVA, Sidak’s multiple comparison test). ^#^ *p* < 0.05, ^##^ *p* < 0.01, ^###^ *p* < 0.001, ^####^ *p* < 0.0001 (expression levels at Day 3, 6, 9, or 12 compared to Day 0, at 33°C or 37°C; one-way ANOVA, Dunnett’s multiple comparison test). Day 0 in both groups is considered as the non-senescence group (control). For clarity, only one-sided error bars are shown.

To further characterize the development of the senescence phenotype in our ciPTEC-OAT1 model, the expression levels of known senescence hallmarks (p53, LaminB1, p21, and β-gal) were evaluated over time at permissive and non-permissive temperatures of 33 and 37°C, respectively (Hernandez-Segura et al., 2018). The obtained results suggest that the expression of these markers in ciPTEC-OAT1 is influenced by maturation in non-permissive conditions of 37°C (Figure 3). The total-p53 levels (Figures 3A,C) were markedly upregulated in a time-dependent manner in the proliferation group at 33°C but presented a decreasing trend of expression in the maturation group at 37°C. The expression levels of total-p53 were different on all days tested between the two groups, with higher levels at 33°C. The expression of LaminB1 (Figures 3B,D) showed a non-significant trend of decreased expression especially after longer culture (Days 9 and 12) at both temperatures, with the most evident trend of different expression between the two groups being at Day 9. Furthermore, the results show that p21 levels (Figures 3E,F) were increased at both 33 and 37°C, and that compared to Day 0, p21 was significantly upregulated on Day 12 at 33°C and on Days 3 through 12 at 37°C. In addition, in non-permissive conditions, there was a significant increase of p21 expression compared to permissive temperature at Days 3 and 6. Regarding β-gal (Figures 3E,G), the protein expression levels show an increasing trend at both 33 and 37°C temperatures, there were significant increases of β-gal expression compared to non-senescent cells (Day 0) at Days 3 and 6 at 37°C, with levels being markedly higher at non-permissive compared to permissive conditions at Day 6.

### 3.4 Maturation of ciPTEC-OAT1 at Non-Permissive Temperature Induces Common SASP Factors Secretion

**FIGURE 4:**
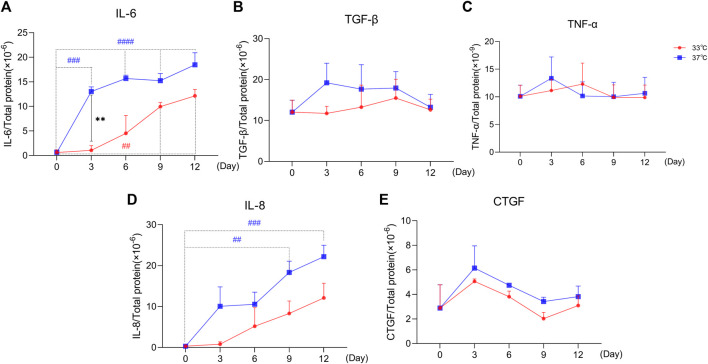
Maturation at a non-permissive temperature of 37°C affects common SASP factors secretion by ciPTEC-OAT1. Release of **(A)** IL-6, **(B)** TGF-β1, **(C)** TNF-α, **(D)** IL-8, and **(E)** CTGF by ciPTEC-OAT1 over time at 33 and 37°C. Concentration is expressed as pg/ml and normalized for total protein (µg/ml). Three independent experiments were performed in triplicate. *p < 0.05, **p < 0.01, ***p < 0.001, ****p < 0.0001 (secreted levels at 37°C compared to 33°C; two-way ANOVA, Sidak’s multiple comparison test). ^#^ *p* < 0.05, ^##^ *p* < 0.01, ^###^ *p* < 0.001, ^####^ *p* < 0.0001 (secreted levels at Day 3, 6, 9, or 12 compared to Day 0, at 33°C or 37°C; one-way ANOVA, Dunnett’s multiple comparison test). Day 0 in both groups is considered as the non-senescence group (control). For clarity, only one-sided error bars are shown.

As shown in Figure 4, the secretion profile of some typical SASP factors (IL-6, TGF-β1, TNF-α, IL-8, and CTGF) clearly correlate with prolonged culture at 37°C, which is indicative of a senescence phenotype. IL-6 is increasingly secreted (Figure 4A) over time, both at 33 and 37°C culture conditions. The secretion of IL-6 was significantly higher at the non-permissive temperature compared to the permissive temperature at Days 3, 6, and 12. The secreted levels of TGF-β1 did not seem to differ between two culture conditions especially at later time points (Figure 4B). The levels of TNF-α (Figure 4C) were not different over time and between the culture conditions. The secretion profile of IL-8 (Figure 4D) showed a time-dependent increase at both temperature conditions, with a higher trend of expression levels at 37°C compared to 33°C. Finally, the CTGF results (Figure 4E) showed an initial increasing trend in secretion, followed by a trend for reduction and return to basal levels starting from Day 3, at both culture conditions. Compared to the permissive culture conditions, the concentration of CTGF at a non-permissive temperature was slightly higher at all time points.

### 3.5 CiPTEC-OAT1 Exhibiting a Senescence-like Phenotype Are Susceptible to Common Senolytics

Our data suggest that ciPTEC-OAT1 cultured progressively in non-permissive conditions obtains a senescence-like phenotype as indicated by some of the most important markers and SASP factors, especially evident after 9 d of culturing. For that reason, this time point was selected to test the effects of senolytics, a class of small molecules that can selectively eliminate senescent cells participating in associated pathways by interfering with anti- and pro-survival signaling pathways (Zhu et al., 2015) For this, navitoclax, dasatinib, and quercetin were employed to first assess the cell viability after exposures at Day 0 or after 9 d of culture.

**FIGURE 5:**
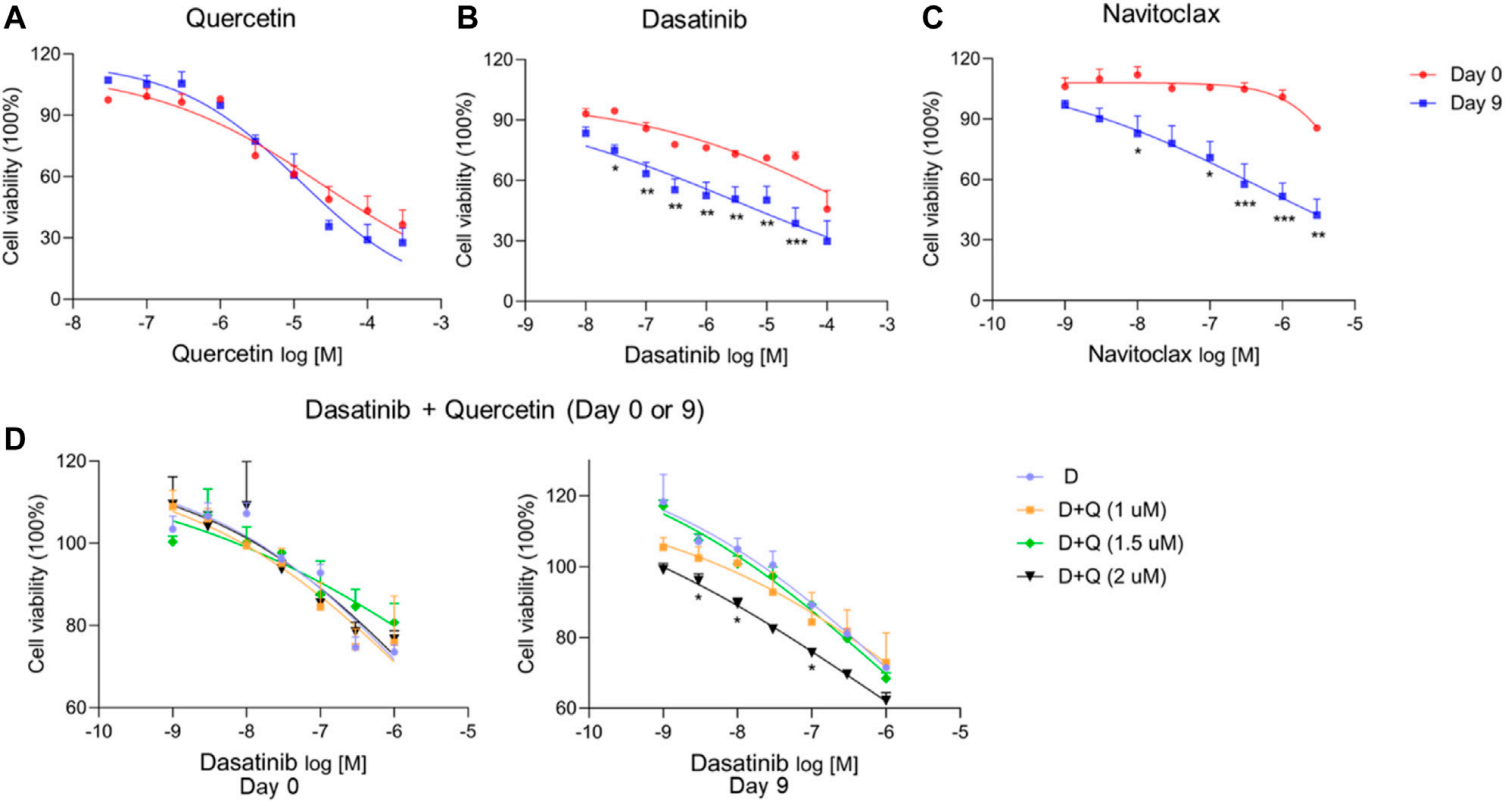
CiPTEC-OAT1 cultured at a non-permissive temperature and exhibiting a senescence-like phenotype is susceptible to common senolytics. Cell viability of ciPTEC-OAT1 cultured for 0 or 9 d at a non-permissive temperature of 37°C and exposed to 100-μl medium with increasing concentrations of **(A)** quercetin (Q), **(B)** dasatinib (D), and **(C)** navitoclax (N) for 24 h. Cell viability of ciPTEC-OAT1 cultured for 0 and 9 d **(D)** at a non-permissive temperature and exposed to 100-μl medium with increasing concentrations of dasatinib combined with quercetin (1 μM, 1.5 μM, or 2 μM) for 24 h. Four independent experiments were performed in triplicate. Data are presented as mean ± SEM, for which results were normalized to unexposed cells. ^*^ *p* < 0.05, ^**^ *p* < 0.01, ^***^ *p* < 0.001 (A to C, cell viability at Day 9 compared to Day 0; two-way ANOVA, Sidak’s multiple comparison test. D, cell viability of increasing concentrations of dasatinib combined with quercetin (1 μM, 1.5 μM, or 2 μM) compared to dasatinib alone; two-way ANOVA, Dunnett’s multiple comparisons test). Day 0 is considered as the non-senescence group (control). For clarity, only one-sided error bars are shown.

As shown in Figure 5; Table 2, quercetin (Figure 5A) did not significantly affect the viability of senescent cells (Day 9 of culture) compared to non-senescent cells (Day 0), while dasatinib (Figure 5B) and navitoclax (Figure 5C) were more effective in selectively reducing the viability of senescent cells at Day 9 compared to Day 0 and therefore can selectively target ciPTEC-OAT1 at a non-permissive temperature. The combination of dasatinib and quercetin (Figure 5D) induced cell death in both culture conditions. The concentrations of quercetin used in this combination treatment are non-toxic to the cells regardless of culture conditions. However, after combining quercetin with increasing concentrations of dasatinib, the viability of the cells reduced compared to each single treatment. Notably, dasatinib combined with the highest quercetin dose appeared the most effective in reducing cell viability. This suggests that the co-treatment of dasatinib and quercetin can selectively target ciPTEC-OAT1 presenting senescence-like phenotype (Figure 5D). IC50 values of different senolytics are lower at Day 9 of cell maturation at a non-permissive temperature compared to 0 d (Table 2). Finally, the cells cultured for 0 or 7 d at 33°C were shown not to be sensitive to navitoclax in contrast to cells cultured at 37°C for 7 d (Supplementary Figure S2), which suggests that the cells are senescent when cultured at 37°C for prolonged time (7 d or more).

**TABLE 2:** IC_50_ of (A)-(D) from Figure 5.

				D + Q			
IC50	Q	D	N	D(Q = 0 μM)	D(Q = 1 μM)	D (Q = 1.5 μM)	D(Q = 2 μM)
Day 0	>10 μM	>3 μM	>3 μM	>3 μM	>4 μM	>3 μM	>4 μM
Day 9	>3 μM	>1 μM	>0.4 μM	>1 μM	>6 μM	>1 μM	>0.7 μM

### 3.6 Senolytics Clear Senescent ciPTEC-OAT1 as Evaluated by Functional SA-β-Gal Expression

**FIGURE 6:**
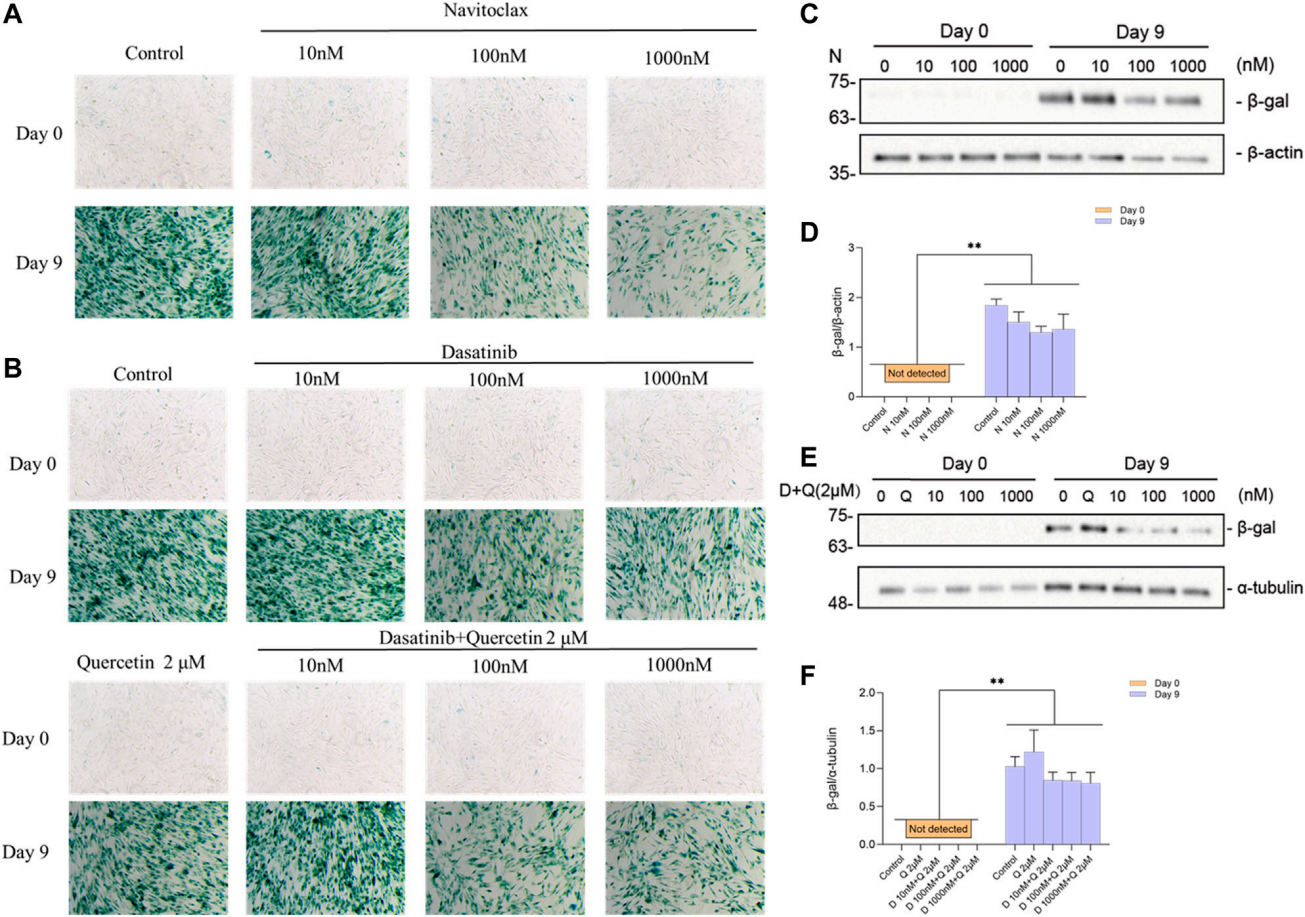
Effects of senolytics on SA-β-gal activity and protein levels in ciPTEC-OAT1 cultured for 9 d at a non-permissive temperature. Representative images of SA-β-gal staining in ciPTEC-OAT1 cultured for 0 and 9 d at 37°C and after 24 h exposure to 1-ml medium with different concentrations of **(A)** navitoclax (N) (10 nM, 100 nM, or 1000 nM) and **(B)** dasatinib (D) alone (10 nM, 100 nM, or 1,000 nM) or combined with quercetin (Q) 2 μM. **(C)** Representative Western blots showing expression of total β-gal after 24 h exposure to different concentrations of navitoclax in ciPTEC-OAT1 cultured for 0 or 9 d at a non-permissive temperature. **(D)** Relative expression of total β-gal after 24 h exposure to different concentrations of navitoclax. **(E)** Representative Western blots showing expression of total β-gal after 24 h exposure to different concentrations of dasatinib combined with quercetin 2 μM in ciPTEC-OAT1 cultured for either 0 or 9 d at a non-permissive temperature. **(F)** Relative expression of total β-gal after 24 h exposure to different concentrations of dasatinib and quercetin 2 μM. Protein expression levels were normalized to α-tubulin or β-actin, and expressed as mean ± SEM. Three independent experiments were performed in triplicate. ^*^ *p* < 0.05, ^**^ *p* < 0.01 (expression levels at 37°C compared to 33°C at the same time point; multiple *t*-test, Holm-Sidak multiple comparison test). Day 0 is considered as the non-senescence group (control). For clarity, only one-sided error bars are shown.

The activity of the lysosomal senescence-associated beta-galactosidase (SA-β-gal) is commonly used as a marker for senescent cells, reflecting increased metabolic activity and enhanced lysosomal content typical of these cells (Hernandez-Segura et al., 2018). We tested the senolytics for their effect on β-gal activity and expression levels. Representative images of SA-β-gal staining upon culturing are shown in Supplementary Figure S3, which gives an overview of senescence process during the maturation of ciPTEC-OAT1 cells. The results obtained (Figures 6A,B) show that ciPTEC-OAT1 cultured for 9 d at 37°C is positive for SA-β-gal staining and that total β-gal protein was increased. Moreover, following exposure to navitoclax, the number of SA-β-gal positive cells was reduced in a dose-dependent fashion (Figure 6A). The expression levels of total β-gal showed a downregulation trend in the presence of navitoclax, with a concentration of 100 nM being the most effective (Figures 6C,D). Furthermore, the number of SA-β-gal positive cells was also reduced dose-dependently after the treatment with both dasatinib alone and dasatinib-quercetin combination, which seemed to be slightly more effective than dasatinib alone (Figure 6B), accompanied by a downregulation of total β-gal (Figures 6E,F). Interestingly, although a lower number of SA-β-gal positive cells was observed after the exposure to higher doses of dasatinib-quercetin combination, there were no differences in protein levels in cells cultured for 9 d at the non-permissive temperature.

## 4 Discussion

Cellular senescence is an irreversible condition with cell cycle arrest, SASP, and apoptosis resistance, which contribute to chronic kidney disease, leading to fibrosis (Stenvinkel and Larsson, 2013; Hernandez-Segura et al., 2018). We previously developed ciPTEC-OAT1 to be used in drug screening and nephrotoxicity studies (Nieskens et al., 2016). In the present study, we demonstrate that the cell model is also suitable for studying tubular senescence in the kidney. We detected differential expression of apoptosis-associated markers, common senescence markers, and some typical SASP factors suggesting that ciPTEC-OAT1 obtains a senescence-like phenotype when cultured at a non-permissive temperature for 9 d. Furthermore, senescent cells appeared sensitive to senolytic drugs.

In the present study, we demonstrated that the ciPTEC-OAT1 cultured at a non-permissive temperature expressed common senescence markers. In particular, the decrease in Lamin B1 (Shimi et al., 2011) and upregulation in p21 (Calcinotto et al., 2019) have been described as characteristic features that are involved in maintaining senescence phenotype by regulating JNK and caspase signaling (Yosef et al., 2017). Further, increased SASP factors have been reported as proinflammatory and matrix-degrading molecules (Childs et al., 2015), including PAI-1 (Sun et al., 2019), IL-1β (Shi et al., 2019), CTGF (Jun and Lau, 2017), and IL-6 (Mosteiro et al., 2018). Another feature entails apoptosis, responsible for cell turnover and maintaining extracellular environment. For instance, the Bcl-2 family members modulate the delicate balance between pro- and anti-apoptosis (Ngoi et al., 2020). The upregulations of Bcl-2 and Bcl-xl suggest that ciPTEC-OAT1 became anti-apoptotic upon maturation in both conditions, whereas culturing at 37°C speeds up the process and induces a more prominent senescence phenotype. Senescent cells show downregulation of Mcl-1 on protein levels (Lee et al., 2018), which is consistent with our findings. Furthermore, the effectors Bax and Bak shuttle between cytosol and mitochondrial outer membrane with different rates (Pena-Blanco and Garcia-Saez, 2018), which might explain why we observed a differential expression in the proteins. BH3-only proteins bind to the BH3 domain of the anti-apoptotic Bcl-2 proteins via hydrophobic interactions, thereby promoting cellular apoptosis (Anantram and Degani, 2019). Of BH3-only proteins, Bid showed higher protein levels at different time points in the maturation group. The increasing trend in all other proteins points toward a priming of cells to undergo apoptosis, but the execution of the death program is restrained. These findings are in line with previous reports showing that following senescence induction by ionizing radiation, senescent cells upregulate pro-apoptosis markers (Chang et al., 2016; Baar et al., 2017). Therefore, cellular senescence in our model with an upregulation of pro-apoptotic markers despite having an anti-apoptotic phenotype argues for cells searching for a new balance to maintain homeostasis.

**FIGURE 7:**
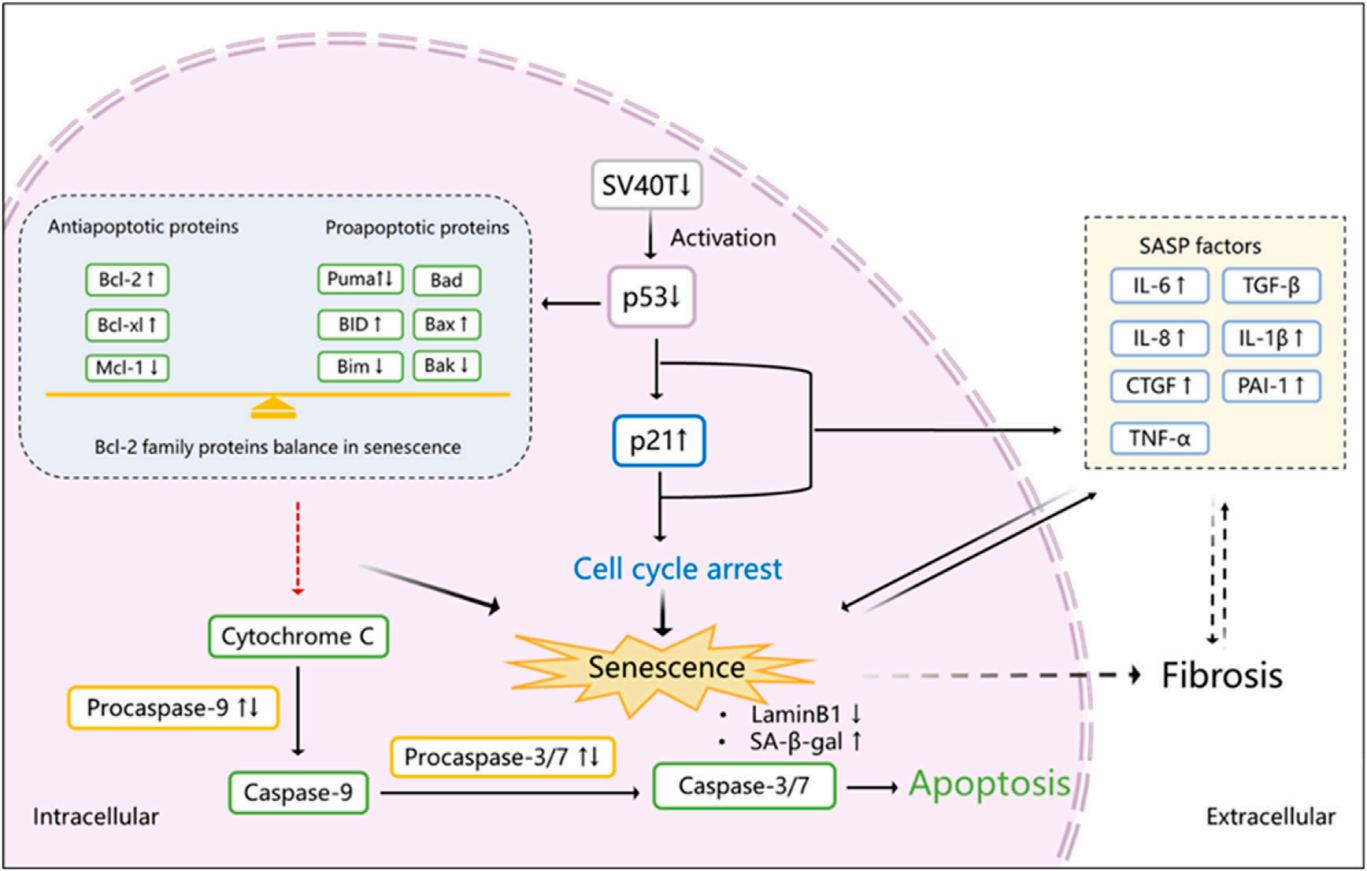
Proposed scheme of senescence induction in ciPTEC-OAT1 after maturation at a non-permissive temperature. After transfer of cells to 37°C and subsequent downregulation of SV40T, p53 is activated which transcriptionally upregulates p21, inducing cell cycle arrest and eventually leading to senescence. SASP factors are released during this process as well. Activated p53 also influences the expression of Bcl-2 family proteins that become abnormally upregulated. Procaspase 9 and its downstream proteins procaspases 3 and 7 are activated at the beginning of this process, and with time going by, the Bcl-2 family reaches a balance between anti-apoptotic and pro-apoptotic proteins expression, halting the activation of procaspases and inhibiting apoptosis. In addition, senescent cells show a downregulation of LaminB1 and an upregulation of SA-β-gal and some SASP factors, including IL-6, IL-8, CTGF, IL-1β, and PAI-1, which may further contribute to kidney fibrosis. ↑, upregulation; ↓, downregulation; ↑↓, initial upregulation followed with downregulation (expression levels at 37°C compared to 33°C)*.*

Caspases are another group of proteins involved in cell death mediated by apoptosis and important senescent markers (Shalini et al., 2015). After MOMP, caspase activation takes place often within minutes, leading to cell death (Pena-Blanco and Garcia-Saez, 2018). Inhibition of caspases therefore blocks apoptosis. Here we detected that the activator (procaspase-9) and executioner (procaspase-3 and procaspase-7) were upregulated in a permissive temperature group but show a differential pattern when cells are cultured at the non-permissive temperature. When mitochondrial-mediated apoptosis is induced and caspase-9 and caspase-3 are activated, the expression of Bax and Bcl-2 has been reported to show different levels to maintain their balance (Zhang et al., 2021). Irradiation-induced senescence is accompanied by an upregulation of procaspase-3, -7, and -9 (Chang et al., 2016; Baar et al., 2017) but a downregulation of activated caspase-3 (Chang et al., 2016), in line with our results. The final downregulation of procaspases observed in our study might be explained by the cleavage of procaspase-9 finally to active caspase-3 and caspase-7. But because of senescence induction and an adapted balance in Bcl-2 family proteins, procaspase-9 is inhibited, finally leading to apoptosis-resistance (Figure 7).

The observed loss of LaminB1 and upregulation of β-gal, well-known indicators of senescence (Hernandez-Segura et al., 2018), in ciPTEC-OAT1 cultured at a non-permissive temperature further confirms the phenotypical changes. Silencing LaminB1 immediately leads to inhibition of proliferation and the induction of senescence (Shimi et al., 2011). Our previous research regarding cell cycle analysis of ciPTEC-OAT1 at permissive and non-permissive temperatures (Mihajlovic et al., 2019) has shown that ciPTEC-OAT1 when cultured at 37°C for 1 or 7 d exhibits significantly reduced proliferation (less cells in the S phase) and an increased number of cells in the G0/G1 phase of the cell cycle, indicating halted proliferation at a non-permissive temperature. This is in line with our current results. The activity of the lysosomal β-gal reflects increased metabolic activity and enhanced lysosomal content typical of senescent cells (Hernandez-Segura et al., 2018). Transfection with the temperature sensitive SV40T gene allows the cells to become conditionally immortalized (Wilmer et al., 2010). Although downregulation of SV40T at 37°C allows activation of p53 and pRb, factors involved in both p53/p21 and p16/pRb pathways, there was no significant difference in their mRNA expression (Supplementary Figure S1) and p16 protein expression appeared undetectable. This suggests that senescence of ciPTEC-OAT1 may not be induced by the p16/pRb pathway. On the other hand, p53 is pivotal in determining the fate of the cells, implying that the p53/p21 pathway is key in the initiation of senescence (Mijit et al., 2020). Previously published works described a decline of total-p53 levels in stress-induced senescence in mice (Feng et al., 2007) and p21’s role in maintaining senescence in mice (Yosef et al., 2017), which is also in accordance with our *in vitro* data showing a decline in p53 levels and an increase in p21 levels at 37°C. Therefore, our results suggest that ciPTEC-OAT1 cultured at 37°C promotes senescence through the p53/p21 pathway.

In addition to previously tested markers, SASP factors are also important players in senescence. IL-6 maintains senescence through the p53/p21 pathway (Effenberger et al., 2014; Li Y. et al., 2020), shared by IL-8, which is expressed as a function of IL-6 (Kuilman et al., 2008). TGF-β1 and CTGF are other SASP factors reported to mediate senescence (Jun and Lau, 2017; You et al., 2019). CTGF is a downstream mediator of TGF-β1 and is regulated by TGF-β1 (Ou et al., 2020). TGF-β1 induces kidney fibrosis by accumulation of extracellular matrix and CTGF expression by activation of Smad3 and p53 (Li X. et al., 2020). Meanwhile, both CTGF and TGF-β1 induce senescence and are accompanied with the upregulation of IL-6 and IL-8 (Jun and Lau, 2017; Fan et al., 2019). Although not significant, our results show an increasing trend of both TGF-β1 and CTGF in 37°C group compared to 33°C group. PAI-1 is a major TGF-β1/p53 target gene in kidney fibrosis and is known to be elevated in senescent cells, correlating with increased tissue TGF-β1 levels (Samarakoon et al., 2019; Rana et al., 2020). In our study, PAI-1 and CTGF increased remarkably on the mRNA level, suggesting cellular senescence. TNF-α is another SASP factor and inducer of senescence (Guo et al., 2019), but our results showed no important differences over time and between the culture conditions, indicating the senescence of ciPTEC-OAT1 is not induced or maintained by TNF-α. There are some discrepancies between mRNA and protein levels of the obtained results. Despite being difficult to explain, these discrepancies might be due to differences in the regulation of transcription and protein translation processes, as well as protein turnover rate. Taken together, the results of the mRNA and protein levels, we believe that the cells exhibit a senescence phenotype at a non-permissive temperature.

Finally, the senolytics navitoclax, dasatinib, and quercetin were evaluated. Our results suggest that ciPTEC-OAT1 cultured at a non-permissive temperature was sensitive to senolytics, and the Bcl-2 family inhibitor navitoclax (Tse et al., 2008; Chang et al., 2016), navitoclax, appeared most effective in selectively reducing viability of cells presenting senescent phenotype. A clinical trial of the dasatinib and quercetin cocktail demonstrated a decrease in p21 and p16 positive human adipose tissue cells and plasma SASP factors of diabetic kidney disease participants (Hickson et al., 2019). Senolytics treatment of ciPTEC-OAT1 led to a dose-dependent reduction of SA-β-gal positive cells, in line with previous results (Zhu et al., 2015). It has been suggested that SA-β-gal activity may be an outcome rather than a cause of senescence (Lee et al., 2006; Piechota et al., 2016), and our findings argue for a clearance of senescent cells leading to a reduction in total cell number. Our follow-up research will focus on investigating the underlying mechanisms of senolytics used in this study.

In conclusion, our results suggest ciPTEC-OAT1 can be used as a valid proximal tubule cell model both for mechanistic studies inherent to kidney senescence and fibrosis and for senolytic effects of newly developed drugs and their combinations.

## Data Availability

The raw data supporting the conclusions of this article will be made available by the authors, without undue reservation.
