# Supplementary material for: A Human Conditionally Immortalized Proximal Tubule Epithelial Cell Line as a Novel Model for Studying Senescence and Response to Senolytics
Source: Front Pharmacol. 2022 Mar 29;13:791612. doi: 10.3389/fphar.2022.791612 (PMC9002109; doi:10.3389/fphar.2022.791612)
Supplement: Supplementary file 1 [file DataSheet1.PDF]

Supplementary Material

A human conditionally immortalized proximal tubule epithelial cell line as a novel model for studying senescence and response to senolytics

Yi Yang[1], Milos Mihajlovic[1], Floris Valentijn[2], Tri Q. Nguyen[2], Roel Goldschmeding[2] and Rosalinde Masereeuw[1,*]

[1]*Utrecht Institute for Pharmaceutical Sciences, div. Pharmacology, Utrecht, The Netherlands.*

[2]*University Medical Center Utrecht, Dept. Pathology, Utrecht, The Netherlands.*

Correspondence*:

Rosalinde Masereeuw

R.Masereeuw@uu.nl

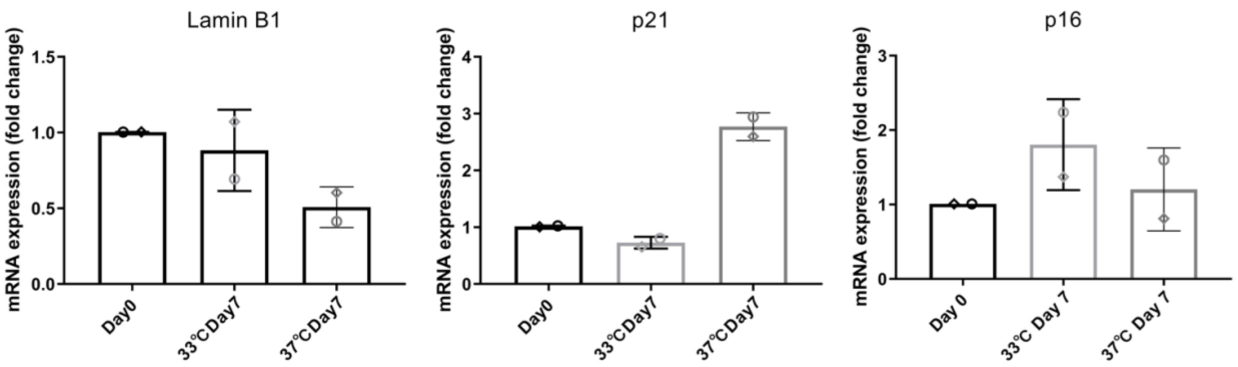

Figure S1. Gene expression levels of Lamin B1, p21 and p16 at permissive and non-permissive temperatures. mRNA levels of LaminB1, p21 and p16 detected after culturing ciPTEC-OAT1 for 0 or 7 days at permissive (33 °C) and non-permissive temperature (37 °C). Two independent experiments in triplicates were performed.

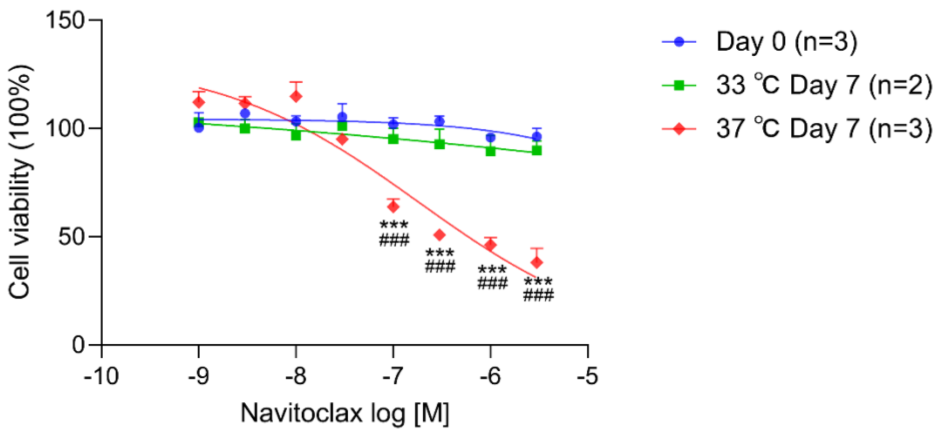

Figure S2. CiPTEC-OAT1 cultured at non-permissive temperature and exhibiting a senescence-like phenotype are susceptible to navitoclax. Cell viability of ciPTEC-OAT1 cultured for 0 or 7 days at permissive (33 °C) and non-permissive temperature (37 °C) and exposed increasing concentrations of navitoclax. At least two independent experiments in

triplicates were performed. Data are presented as mean ± SEM, for which results were normalized to unexposed cells. *P<0.05, **P<0.01, ***P<0.001 (cell viability at 37℃ Day 7 compared to Day 0 at the same concentration; Multiple t-test, Holm-Sidak multiple comparison test). #P<0.05, ##P<0.01, ###P<0.001 (cell viability at 37℃ Day 7 compared to 33℃ Day 7 at the same concentration; Multiple t-test, Holm-Sidak multiple comparison test).

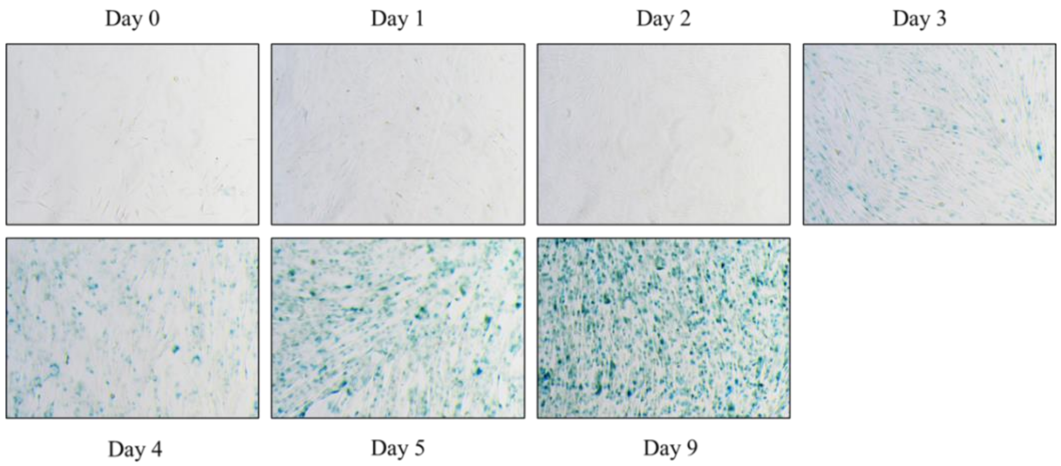

Figure S3. SA-β-gal activity in CiPTEC-OAT1 during culture at non-permissive temperature. Representative images of SA-β-gal staining in ciPTEC-OAT1 cultured for 0 through 9 days at 37 ℃. Three independent experiments were performed.
